# Interstitial Lung Abnormalities Detected by CT in Asbestos-Exposed Subjects Are More Likely Associated to Age

**DOI:** 10.3390/jcm10143130

**Published:** 2021-07-15

**Authors:** François Laurent, Ilyes Benlala, Gael Dournes, Celine Gramond, Isabelle Thaon, Bénédicte Clin, Patrick Brochard, Antoine Gislard, Pascal Andujar, Soizick Chammings, Justine Gallet, Aude Lacourt, Fleur Delva, Christophe Paris, Gilbert Ferretti, Jean-Claude Pairon

**Affiliations:** 1 Faculté de Médecine, Université de Bordeaux, F-33000 Bordeaux, France; ilyes.ben-lala@u-bordeaux.fr (I.B.); gael.dournes@u-bordeaux.fr (G.D.); patrick.brochard@chu-bordeaux.fr (P.B.); 2 Service d’Imagerie Médicale Radiologie Diagnostique et Thérapeutique, CHU de Bordeaux, F-33000 Bordeaux, France; 3 Centre de Recherche Cardio-Thoracique de Bordeaux, INSERM U1045, Université de Bordeaux, F-33000 Bordeaux, France; 4 Epicene Team, Bordeaux Population Health Research Center, INSERM UMR 1219, Université de Bordeaux, F-33000 Bordeaux, France; celine.gramond@u-bordeaux.fr (C.G.); justine.gallet@u-bordeaux.fr (J.G.); aude.lacourt@inserm.fr (A.L.); fleur.delva@chu-bordeaux.fr (F.D.); 5 Centre de Consultation de Pathologies Professionnelles, CHRU de Nancy, Université de Lorraine, F-54000 Nancy, France; i.thaon@chru-nancy.fr; 6 Service de Santé au Travail et Pathologie Professionnelle, CHU Caen, F-14000 Caen, France; clin-b@chu-caen.fr; 7 Faculté de Médecine, Université de Caen, ANTICIPE, INSERM U1086, F-14000 Caen, France; 8 Service de Médecine du Travail et de Pathologies Professionnelles, CHU de Bordeaux, F-33000 Bordeaux, France; 9 Centre de Consultations de Pathologie Professionnelle, UNIROUEN, UNICAEN, ABTE, F-76000 Rouen, France; Antoine.Gislard@chu-rouen.fr; 10 CHU de Rouen, Normandie Université, F-76031 Rouen, France; 11 Equipe GEIC20, INSERM U955, F-94000 Créteil, France; pascal.andujar@inserm.fr (P.A.); jc.pairon@chicreteil.fr (J.-C.P.); 12 Faculté de Santé, Université Paris-Est Créteil, F-94000 Créteil, France; 13 Service de Pathologies Professionnelles et de l’Environnement, Centre Hospitalier Intercommunal Créteil, Institut Santé-Travail Paris-Est, F-94000 Créteil, France; 14 Institut Interuniversitaire de Médecine du Travail de Paris-Ile de France, F-94000 Créteil, France; soizick.chammings@iimtpif.fr; 15 Service de Santé au Travail et Pathologie Professionnelle, CHU Rennes, F-35000 Rennes, France; christophe.paris@inserm.fr; 16 Institut de Recherche en Santé, Environnement et Travail, INSERM U1085, F-35000 Rennes, France; 17 INSERM U 1209 IAB, F-38700 La Tronche, France; GFerretti@chu-grenoble.fr; 18 Domaine de la Merci, Université Grenoble Alpes, F-38706 La Tronche, France; 19 Service de Radiologie Diagnostique et Interventionnelle Nord, CHU Grenoble Alpes, CS 10217, F-38043 Grenoble, France

**Keywords:** asbestos-exposition, HRCT, asbestosis

## Abstract

Objective: the aim of this study was to evaluate the association between interstitial lung abnormalities, asbestos exposure and age in a population of retired workers previously occupationally exposed to asbestos. Methods: previously occupationally exposed former workers to asbestos eligible for a survey conducted between 2003 and 2005 in four regions of France, underwent chest CT examinations and pulmonary function testing. Industrial hygienists evaluated asbestos exposure and calculated for each subject a cumulative exposure index (CEI) to asbestos. Smoking status information was also collected in this second round of screening. Expert radiologists performed blinded independent double reading of chest CT-scans and classified interstitial lung abnormalities into: no abnormality, minor interstitial findings, interstitial findings inconsistent with UIP, possible or definite UIP. In addition, emphysema was assessed visually (none, minor: emphysema <25%, moderate: between 25 and 50% and severe: >50% of the lung). Logistic regression models adjusted for age and smoking were used to assess the relationship between interstitial lung abnormalities and occupational asbestos exposure. Results: the study population consisted of 2157 male subjects. Interstitial lung abnormalities were present in 365 (16.7%) and emphysema in 444 (20.4%). Significant positive association was found between definite or possible UIP pattern and age (OR adjusted =1.08 (95% CI: 1.02–1.13)). No association was found between interstitial abnormalities and CEI or the level of asbestos exposure. Conclusion: presence of interstitial abnormalities at HRCT was associated to aging but not to cumulative exposure index in this cohort of former workers previously occupationally exposed to asbestos.

## 1. Introduction

The development and severity of asbestosis is related to intensity of exposure to asbestos and time since first exposure [1]. The surveillance of the former exposed workers is justified by financial compensations and because of the elevated risk of bronchial cancer. Therefore, and because of the long latency of asbestosis, health surveillance should be prolonged after the exposure. High resolution computed tomography (HRCT) is able to detect asbestos-induced pulmonary changes much earlier than chest x-ray and is useful for early diagnosis of asbestosis [2,3,4]. However, today, the prevalence of asbestosis is lower than in studies of past decades [5,6]. In addition, the pattern of asbestosis has changed and mild fibrosis has been reported [6,7]. Interstitial lung abnormalities (ILA) are defined as early interstitial changes in nondependent areas of the lung, and has been validated as evidence of subclinical interstitial lung disease (ILD) [8]. Subjects with subclinical ILD exhibit more respiratory symptoms, physiologic decline and higher mortality [9,10]. However, in a population of middle-aged to elderly subjects, ILA have been associated with a mild form of pulmonary fibrosis in smokers and non-smokers [8,10] and has been reported to increase with age [11,12,13,14]. Moreover, longitudinal studies have shown progression of imaging patterns of ILA [9] and that have been related to mortality [15]. Indeed, a recent study showed that the prevalence of HRCT patterns of usual interstitial pneumonia (UIP) and chronic interstitial pneumonia were 0.3% and 3.8%, respectively, in a smokers’ cohort with 25% of progression in those who underwent a 3 year follow-up CT scan [13]. Therefore, the relationships between age, asbestos-exposure and smoking status need to be clarified.

The present study was designed to evaluate the association between interstitial abnormalities, asbestos exposure and age in a population of former workers previously occupationally exposed to asbestos. The main objective of this study was to determine whether ILA identified in a population of former asbestos-exposed workers were due to asbestosis or also to other causes of interstitial lung disease such age and/or smoking.

## 2. Material and Methods

### 2.1. Study Population

A first round of a screening program for asbestos-related diseases was held between October 2003 and December 2005 in four regions of France. Retired workers exposed to asbestos during their working life and without already compensated asbestos-related disease were eligible in this surveillance program. As previously described, volunteers were invited to participate using different ways and constituted the Asbestos-Related Diseases Cohort (ARDCO). They could beneficiate of a free medical check-up including chest CT-scan and pulmonary function tests [1,16,17,18]. Subjects having CT-scan sent to the regional coordinating centers constituted the Asbestos Post-Exposure Survey (APEXS) population. All male subjects of the APEXS population were included in the present study. A second round of screening was organized 5 years later and this study is based on the second round. Indeed, digitalized thin-section CT examinations of the second round of the survey showed better image quality than CT exams of the first round where a number of digitalized examinations were missing.

The study was approved by the hospital ethics committee (CPPRB Paris-Cochin n 1946 (2002), CPP Ile De France III n 1946/11/02-02 (2010)). All participants received information about the study and gave their written informed consent.

### 2.2. Asbestos Exposure and Smoking Status

All subjects completed a standardized questionnaire, having different parts: all successive jobs (date of beginning and end, location of the employer, main activity of the firm, precise job of the subject), indication of having ever worked in a list of specific jobs (known for high probability of exposure to asbestos) in the construction industry with indication of date of beginning and end of these jobs; 9 specific questions on tasks entailing exposure to asbestos with indication of date of beginning and end of each task; and finally free text to invite the subject to add any precision he would prefer to add to his previous answers. Therefore, industrial hygienists could evaluate asbestos exposure based on of the complete working life history of each subject. For each job considered exposed to asbestos, the duration (expressed in years) and dates of exposure were determined. The maximum level of exposure was defined as the highest exposure occurring during the entire work history. The following weighting factors were decided for the intensity level of exposure, based on the knowledge of the different situations of exposure: low (passive exposure): 0.01; low intermediate: 0.1; high intermediate: 1; high: 10. A cumulative exposure index (CEI) to asbestos was then calculated for each subject over his working life. It was calculated by summing the exposures calculated for each exposed job (duration x weighting factor). There were no atmospheric measurements in this cohort, and detailed information on the frequency of exposure (percentage of the working time) was frequently lacking. Therefore, the CEI was expressed in exposure units x years rather than fibers/mL x years. The elapsed time between the beginning of the first job considered to be exposed to asbestos and the date of CT scan was calculated as the time since first exposure (TSFE) [16,17,18].

The questionnaire also collected information on smoking status, allowing classification of subjects into three categories: current smokers, ex-smokers (those who had stopped smoking for at least one year) and never-smokers (having smoked less than 100 cigarettes over the working life).

### 2.3. CT Scanning

A specific protocol was established by a group of chest radiologists as previously described [1]. Four experts trained in the interpretation of asbestos-related CT abnormalities performed a double-blinded independent evaluation of all CT examinations. Triple evaluation was performed in the case of disagreement between the first two readings. These evaluations were performed in a blinded fashion to the subject’s cumulative exposure to asbestos and to the report of the initial reading made by the radiologists who performed the CT examination. Interstitial or pleural abnormalities were registered using a standardized form according to the Fleischner Society glossary of terms [19].

The reader was asked to classify the patient into one of four categories for parenchymal findings: no abnormality, minor interstitial findings, interstitial findings inconsistent with UIP, possible or definite UIP. The second category was based on the description of abnormal interstitial findings in aging people [11] and the 2 last categories based on the consensus ATS/ERS criteria for the diagnosis of idiopathic pulmonary fibrosis (IPF) [20]. Severity of emphysema was assessed visually on a 4-level scale (none, minor: emphysema occupying less than 25%, moderate: between 25 and 50% and severe: more than 50% of the whole pulmonary volume).

### 2.4. Statistical Analysis

The relationship between interstitial lung abnormalities and occupational asbestos exposure was estimated using logistic regression models adjusted for age and smoking (never smokers, ex-smokers and current smokers). Occupational asbestos exposure was characterized by the CEI, time since first exposure and the maximum level of exposure. Linearity of quantitative variables was examined using fractional polynomial [21]. Supplementary analyses were conducted to study the association between severity emphysema at CT and occupational asbestos exposure using the same analysis strategy as described above. Statistical analyses were carried out using SAS software version 9.4 (SAS Institute, Inc, Cary, NC, USA) and R software version 3.4.2.

## 3. Results

### 3.1. Subjects’ Demographic Data, Smoking Data, Asbestos Exposure and Frequency of Interstitial Abnormalities at CT

**Figure 1:**
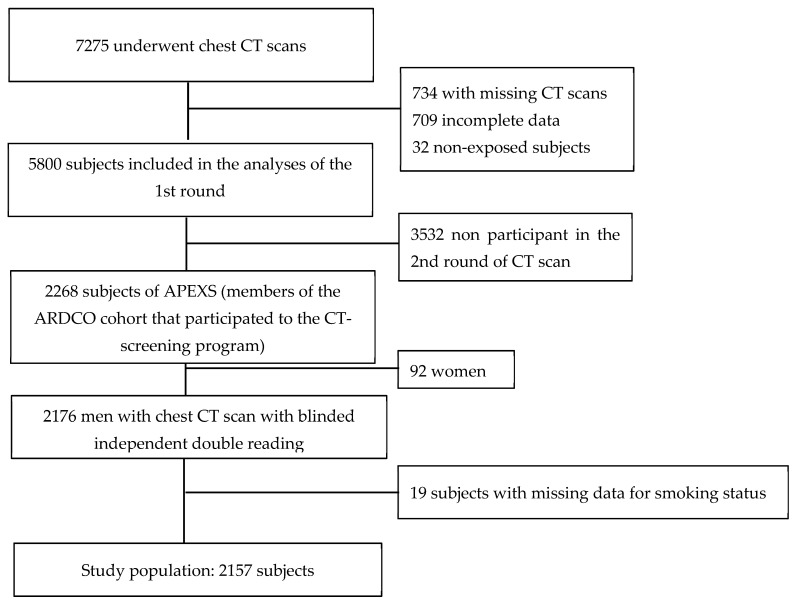
Study Flow-Chart. APEXS: Asbestos Post-Exposure Survey; ARDCO: Asbestos-Related Diseases Cohort; CT = computed tomography.

At the second round of the screening program proposed to the APEXS population, 2268 subjects have been explored by CT with a double readings and triple reading if discordances. After exclusion of 92 women and 19 subjects with missing data for smoking status, the study population consisted of 2157 male subjects (Figure 1). Pleural plaques were present in 559 (25.7%) subjects, interstitial abnormalities in 365 (16.7%), emphysema in 444 (20.4%) (Table 1).

### 3.2. Association between Possible and Definite CT Patterns of UIP and Age, Smoking Status and the Level of Exposure to Asbestos

The frequency of interstitial findings according to demographic data, smoking status and asbestos exposure is shown Table 2.

A significant positive association was found between UIP pattern or possible UIP pattern and age (OR _crude_ = 1.08 (95% CI: 1.03–1.13) and OR _adjusted_ = 1.08 (95% CI: 1.02–1.13)) for each additional year (Table 3).

In addition, UIP pattern or possible UIP pattern were significantly associated to smoking status (OR _crude_ for ex-smoker = 2.13 (95% CI: 1.03–4.39)) (Table 3).

There was no significant association between interstitial lung abnormalities and asbestos exposure assessed either by CEI or by maximum level of exposure (Table 3). We have also combined the two intermediate categories of maximum level of exposure (intermediate low + intermediate high) and the results were not significant (data not shown).

When patients with either definite UIP pattern or possible UIP pattern and inconsistent with UIP pattern were grouped, this association with age remained significant (OR _crude_ = 1.09 (95% CI: 1.06–1.12) and OR _adjusted_ = 1.09 (1.05–1.13)) (Supplementary Table S1). On the other hand, the association with asbestos exposure remained non-significant (Supplementary Table S1).

### 3.3. Association between CT Severity of Emphysema and Age, Smoking Status and the Level of Exposure to Asbestos

The frequency of emphysema findings according to demographic data, smoking status and asbestos exposure is shown in Table 4. No association was found between emphysema and CEI to asbestos, but an association was observed with the maximum level of exposure to asbestos (OR _crude_ = 2.05 (95% CI: 1.06–3.99) and OR _adjusted_ = 2.27 (95% CI: 1.08–4.80) for subjects with “high-level” of asbestos exposure). As expected, a significant association was found between emphysema and smoking status (Supplementary Table S2).

## 4. Discussion

This study has shown the association between interstitial abnormalities and age after adjustment on smoking status and asbestos exposure in a population of retired workers previously occupationally exposed to asbestos. This result is consistent with the CT lung cancer screening study by Vehmas et al. [22] among asbestos-exposed workers, where a positive correlation was found between interstitial lung abnormalities at CT and aging after adjustment to smoking status, asbestos exposure and body mass index.

The strengths of our study are the large number of subjects, individual estimation of cumulative occupational exposure to asbestos, HRCT acquisition and accurate analysis of CT by experts and categorization of interstitial abnormalities according to the ATS/ERS consensus criteria [20]. However, our analysis predates international consensus guideline for the diagnosis of IPF [23]. Nevertheless, definitions of UIP patterns are quite similar.

In a population of middle-aged to elderly subjects without occupational exposure, the presence of discrete lung parenchymal abnormalities has been reported to increase with age [11,24]. Jin et al. reported a positive association of fibrotic ILA at HRCT and age [25]. In this case, 60% of healthy subjects aged 75 or older have shown a basal reticular pattern whereas in those younger than 55 years old, no interstitial abnormalities have been reported [11]. This may be due to elastin degradation with aging, which leads to alveolar collapse or to mild interstitial fibrosis. In addition, interlobular septae thickening has been found more commonly in older subjects. However, subpleural lines may disappear at imaging in prone position in some healthy individuals. A study by Gamsu et al. [3] showed that CT findings of asbestosis are neither perfectly sensitive nor specific for asbestosis. In another study by Copley et al., the results of CT evaluation of 74 patients with asbestosis were compared to those of 212 patients with idiopathic pulmonary fibrosis showing that HRCT patterns of asbestosis are closely akin to the UIP pattern [26]. HRCT is today an essential component of the diagnostic pathway in interstitial lung disease. In order to clarify the management of patient with IPF, ATS/ERS guidelines have been published to assess the probability of the disease according to patterns [20].

Asbestosis, however, cannot be distinguished by HRCT from a possible or definite UIP pattern [26]. The first round of screening of this cohort has been reported [1] and conversely to the agreement between trained expert readers for the detection of pleural plaques that was good to excellent, the agreement between trained expert readers for the detection of interstitial abnormalities has been reported to be fair to good [27]. In an attempt to circumvent the subjects with possible mild or moderate asbestosis, we separated those subjects from those with an interstitial pattern inconsistent with UIP, using the criteria defined by ATS/ERS consensus paper [20]. Patterns with upper lobe or middle lung interstitial abnormalities predominance, predominance of ground-glass opacities, nodular pattern, peribronchial pattern, cysts and/or air trapping or mosaic attenuation in three or more lobes were classified globally in the group inconsistent with UIP pattern [20]. Interestingly in these subjects, interstitial abnormalities were not associated with exposure but with age, even when adjusted on smoking status. However, the non-association between interstitial lung abnormalities and asbestos exposure evaluated using time related markers (i.e., duration of exposure and time since the first exposure) could be related to an over-adjustment with age. Nevertheless, the association between interstitial abnormalities with typical or atypical UIP pattern and age has been reported [15,25,26]. Therefore, these subjects may have a preclinical interstitial lung disease rather than asbestosis.

In our study, the association of UIP or possible UIP pattern at HRCT and smoking in univariate analysis is in line with literature showing that tobacco consumption is a risk factor for development of fibrotic lung abnormalities [28]. This finding was not significant in current smokers which could be explained by the low number of current smokers at the time of the study (*n* = 126). In the study by Jin et al., the association between ILA at HRCT and smoking status was found not significant [25]. However, fibrotic ILAs were differentiated into subtypes (i.e., patients with ground glass opacities: *n* = 12, reticulations: *n* = 9 and honeycombing: *n* = 9) which decreased the strength of the statistical analysis. Nonetheless, in the multivariate model when adjusted for occupational asbestos exposure and age, association with cigarette smoking was found not significant.

The relationships between emphysema and asbestos exposure remain unclear. A positive association was found between emphysema and the maximum level of asbestos exposure but not with the CEI to asbestos. Indeed, CEI is considered as a more precise parameter taking into account the total asbestos exposure along the working carrier, which could be more relevant than the maximum level of exposure that could possibly intermingles other associated exposures. In addition, in a population of heavily exposed people to asbestos, Huuskonen et al. [29] have reported an association between emphysema findings and asbestos exposure, after adjustment for age and smoking. They have differentiated, however, emphysema subtypes and used a more detailed score than we did. The causative role of asbestos on emphysema remains to be determined.

Our study had several limitations. Exclusion of already compensated subjects before the first round of this survey may introduce some selection bias. Moreover, this was a voluntary based participation survey thus, motivations of subjects to not participate in the second round were unknown CT images evaluated in this study were acquired at the second round of screening, where all the examinations were stored on digital support for expert analysis in contrast with first round screening examinations that were already reported [1]. Regarding the asbestos exposure evaluation, no atmospheric measurements was performed in this cohort and detailed information on the frequency of exposure was lacking. Therefore, the CEI was expressed in exposure units x years rather than in fibers/mL x years. However, the semi-quantitative analysis of exposure with ordinal classes allowed us to evaluate the association between increasing exposure levels and interstitial lung abnormalities. The low prevalence of asbestosis in this population is in agreement with recent data in the asbestosis epidemiology [5]. However, since this cohort is based on voluntary participation and subjects who have been already compensated for asbestos-related occupational disease before entering the survey were not included, it is likely than a substantial fraction of subjects with overt asbestosis were not included in the study. There was no histological proof of asbestosis in our population. However, the diagnosis is currently made on the basis of HRCT and an appropriate history of asbestos exposure. Indeed, as a consensus, no surgical lung biopsy is needed in these patients. In addition, we have no control group, but ethical considerations prevent the use of radiation exposure due to CT in non-asbestos-exposed subjects.

We have reported that the presence of interstitial abnormalities at HRCT was not associated to the level of exposure in a population of asbestos-exposed subjects, but to aging. This should raise the issue of ILA as an early stage of IPF with a need of an adequate surveillance.

## Figures and Tables

**Table 1:** Frequency of pleural plaques, interstitial abnormalities and emphysema as detected at CT (*n* = 2157).

	*n*	%
**Pleural plaques**		
No	1604	74.4
Yes	553	25.6
**Interstitial abnormalities**		
Absent or gravity-dependent opacities	1794	83.2
Minor interstitial abnormalities	226	10.5
Interstitial pattern inconsistent with UIP	82	3.8
UIP pattern or possible UIP pattern	55	2.5
**Emphysema**		
None	1716	79.6
Minor (less than 25% of lung volume)	281	13.0
Moderate (25% to 50%)	104	4.8
Severe (more than 50% of lung volume)	56	2.6

UIP: usual interstitial pneumonia.

**Table 2:** Frequency of interstitial abnormalities detected at CT according to demographic characteristics, smoking status and asbestos exposure data.

	Absent or Gravity-Dependent Opacities		Minor Interstitial Abnormalities		Interstitial Abnormalities Inconsistent with UIP		UIP Pattern or Possible UIP Pattern	
	*n*	%	*n*	%	*n*	%	*n*	%
**Age (years)**								
Mean (SD)	69.4 (5.4)		71.5 (5.0)		72.4 (6.7)		72.3 (4.9)	
**Smoking status**								
Non-smoker	519	28.9	69	30.5	22	26.8	9	16.4
Ex-smoker	1163	64.8	150	66.4	56	68.3	43	78.2
Smoker	112	6.2	7	3.1	4	4.9	3	5.5
**Maximum Level of Exposure Based on Labor History**								
Low + Low intermediate	518	28.9	50	22.1	20	24.4	17	30.9
High intermediate	843	47.0	127	56.2	43	52.4	17	30.9
High	433	24.1	49	21.7	19	23.2	21	38.2
**CEI to Asbestos (Unit of Exposure x Years)**								
(0–3.3)	352	19.6	37	16.4	12	14.6	11	20.0
(3.3–13.6)	353	19.7	35	15.5	12	14.6	10	18.2
(13.6–32)	362	20.2	53	23.5	22	26.8	9	16.4
(32–64)	373	20.8	59	26.1	20	24.4	12	21.8
(64 and more)	354	19.7	42	18.6	16	19.5	13	23.6
Mean (SD)	60.6 (99.1)		58.0 (90.7)		63.0 (97.3)		72.4 (110.4)	
**Time Since First Exposure (Years)**								
<40	120	6.7	5	2.2	5	6.1	3	5.5
(40–50)	623	34.7	59	26.1	17	20.7	14	25.5
>50	1051	58.6	162	71.7	60	73.2	38	69.1
Mean (SD)	50.2 (7.0)		52.7 (6.14)		52.6 (7.2)		52.4 (7.3)	
**Duration of Exposure to Asbestos (years)**								
<10	84	4.7	7	3.1	2	2.4	4	7.3
(10–20)	161	9.0	15	6.6	6	7.3	5	9.1
(20–30)	298	16.6	39	17.3	7	8.5	7	12.7
(30–40)	829	46.2	107	47.3	46	56.1	25	45.5
>40	422	23.5	58	25.7	21	25.6	14	25.5
Mean (SD)	31.9 (10.1)		32.9 (9.1)		33.9 (8.8)		31.7 (11.3)	

CEI: cumulative exposure index to asbestos.

**Table 3:** Association between the possible or definite UIP pattern at CT, asbestos exposure and age unadjusted and adjusted on age, occupational asbestos exposure and smoking status.

	Univariate Model	Multivariate Models	
	OR (IC95%)	OR (IC95%)	OR (IC95%)
**Duration of exposure (year)**	0.99 (0.97–1.02)	-	-
**Time since the first exposure (year)**	**1.04 (1.00–1.08)**	1.00 (0.96–1.04)	1.00 (0.96–1.04)
**Maximum level of exposure** Low + Low intermediate (*n* = 605) Intermediate high (*n* = 1030) High (*n* = 522)	1 0.58 (0.29–1.15) 1.45 (0.76–2.78)	-	1 0.58 (0.29–1.15) 1.39 (0.71–2.69)
**CEI to asbestos (100 units of exposure x years)**	1.12 (0.88–1.42)	1.09 (0.85–1.40)	-
**Age at the time of CT examination (year)**	**1.08 (1.03–1.13)**	**1.08 (1.03–1.13)**	**1.08 (1.03–1.13)**
**Smoking status** Non-smoker (*n* = 619) Ex-smoker (*n* = 1412) Smoker (*n* = 126)	**1** **2.13 (1.03–4.39)** 1.65 (0.44–6.19)	1 2.03 (0.98–4.21) 1.97 (0.52–7.47)	1 1.92 (0.92–4.00) 1.86 (0.49–7.06)

CEI: cumulative exposure index to asbestos. Bold values indicate statistical significance.

**Table 4:** Frequency of emphysema at CT according to age, smoking status and asbestos exposure data.

	No Emphysema		Minimal Emphysema		Moderate Emphysema (25% to 50%)		Severe Emphysema (More than 50%)	
	*n*	%	*n*	%	*n*	%	*n*	%
**Age (years)**								
Mean (SD)	69.8 (5.4)		70.1 (5.6)		69.2 (5.1)		69.7 (6.2)	
**Smoking Status**								
Non smoker	558	32.5	51	18.1	6	5.8	4	7.1
Ex-smoker	1080	62.9	208	74.0	82	78.8	42	75.0
Smoker	78	4.5	22	7.8	16	15.4	10	17.9
**Maximum Level of Exposure**								
Low + Low intermediate	500	29.1	72	25.6	25	24	8	14.3
High intermediate	816	47.6	135	48.0	55	52.9	24	42.9
High	400	23.3	74	26.3	24	23.1	24	42.9
**CEI to Asbestos (Unit of Exposure x Years)**								
(0–3.3)	334	19.5	54	19.2	16	15.4	8	14.3
(3.3–13.6)	337	19.6	43	15.3	22	21.2	8	14.3
(13.6–32)	341	19.9	61	21.7	26	25.0	18	32.1
(32–64)	373	21.7	64	22.8	21	20.2	6	10.7
(64 and more)	331	19.3	59	21.0	19	18.3	16	28.6
Mean (SD)	60.0 (98.3)		64.2 (100.5)		60.0 (98.7)		68.1 (94.6)	
**Time Since First Exposure (Years)**								
<40	104	6.1	18	6.4	4	3.8	7	12.5
(40–50)	573	33.4	85	30.2	36	34.6	19	33.9
>50	1039	60.5	178	63.3	64	61.5	30	53.6
Mean (SD)	50.6 (7.1)		51.0 (6.6)		50.8 (6.5)		49.8 (7.9)	
**Duration (Years)**								
<10	75	4.4	11	3.9	7	6.7	4	7.1
(10–20)	139	8.1	29	10.3	10	9.6	9	16.1
(20–30)	278	16.2	43	15.3	20	19.2	10	17.9
(30–40)	814	47.4	135	48.0	38	36.5	20	35.7
>40	410	23.9	63	22.4	29	27.9	13	23.2
Mean (SD)	32.2 (9.9)		32.0 (9.6)		31.1 (10.9)		29.2 (11.9)	

CEI: cumulative exposure index to asbestos.

## Data Availability

The data presented in this study are available on request from the corresponding author. The data are not publicly available due to privacy.

## Supplementary Materials

The following are available online at https://www.mdpi.com/article/10.3390/jcm10143130/s1. Table S1: Association between the presence of UIP pattern (definite, possible UIP pattern) and interstitial pattern inconsistent with UIP pattern at CT and asbestos exposure, age, and smoking status. Table S2: Association between emphysema at CT and asbestos exposure. Table S3: Most represented occupations (ISCO 68) in the general population (*n* = 2157) and according interstitial abnormalities detected at CT. Table S4: Most represented industries (ISIC Rev 2) in all subjects and according interstitial abnormalities detected at CT.
