# Supplementary material for: Interstitial Lung Abnormalities Detected by CT in Asbestos-Exposed Subjects Are More Likely Associated to Age
Source: J Clin Med. 2021 Jul 15;10(14):3130. doi: 10.3390/jcm10143130 (PMC8307087; doi:10.3390/jcm10143130)
Supplement: Supplementary file 1 [file jcm-10-03130-s001.zip › jcm-1256116-supplementary.pdf]

Journal of
Clinical Medicine

MDPI

Supplementary Materials

Table S1. Association between the presence of UIP pattern (definite, possible UIP pattern) and interstitial pattern inconsistent with UIP pattern at CT and asbestos exposure, age, and smoking status.

	Univariate Model	Multivariate Models	
	**OR (IC95 %)**	**OR (IC95%)**	**OR (IC95%)**
**Duration of exposure (year)**	1.01 (0.99-1.02)	-	-
**Time since the first exposure (year)**	**1.05 (1.02-1.07)**	1.00 (0.97-1.03)	1.00 (0.97-1.03)
**Maximum level of exposure**			
Low + low intermediate (n=605)	1	-	1
Intermediate high (n=1030)	0.95 (0.62-1.45)		0.95 (0.62-1.46)
High (n=522)	1.27 (0.80-2.02)		1.27 (0.79-2.03)
**CEI to asbestos (100units of exposure x years)**	1.06 (0.90-1.26)	1.05 (0.89-1.24)	-
**Age at the time of CT examination (year)**	**1.09 (1.06-1.12)**	**1.09 (1.05-1.13)**	**1.09 (1.05-1.13)**
**Smoking status**			
Non-smoker (n=619)	1	1	1
Ex-smoker (n=1412)	1.43 (0.94-2.16)	1.37 (0.90-2.09)	1.34 (0.88-2.05)
Smoker (n=126)	1.11 (0.48-2.59)	1.38 (0.59-3.24)	1.35 (0.57-3.16)

CEI: cumulative exposure index to asbestos. Bold values indicate statistical significance.

Table S2. Association between emphysema at CT and asbestos exposure, age, and smoking status.

	Unadjusted OR (IC95 %)	Adjusted OR (IC95%)
**Duration of exposure (years)**	0.99 (0.98-1.00)	-
**Time since first exposure (years)**	1.00 (0.99-1.01)	1.00 (0.98-1.03)
**Maximum level of exposure**		
Low + low intermediate (n=605)	1	1
Intermediate high (n=1,030)	1.25 (0.96-1.62)	1.23 (0.94-1.60)
High (n=522)	**1.45 (1.08-1.94)**	2.30 (0.96-1.76)
**CEI to asbestos (100units of exposure x years)**	1.04 (0.94-1.15)	0.89 (0.75-1.06)
**Age (years)**	1.00 (0.98-1.02)	1.01 (0.98-1.03)
**Smoking status**		
Non-smoker (n=619)	**1**	**1**
Ex-smoker (n=1,412)	**2.81 (2.10-3.76)**	**2.75 (2.05-3.69)**
Smoker (n=126)	**5.63 (3.60-8.79)**	**5.72 (3.63-8.99)**

CEI: cumulative exposure index to asbestos. Bold values indicate statistical significance.

Table S3. Most represented occupations (ISCO 68) in the general population (*n*=2,157) and according interstitial abnormalities detected at CT.

	All Subjects (*n*=2,157)		Absent or Gravity-Dependant Opacities (*n*=1,794)		Minor Interstitial Abnormalities (*n*=226)		Interstitial Abnormalities Inconsistent with UIP (*n* =82)		UIP Pattern or Possible UIP Pattern (*n*=55)	
	n	%	*n*	%	*n*	%	*n*	%	*n*	%
8-49 Machinery Fitters, Machine Assemblers and Precision-Instrument Makers [except Electrical] Not Elsewhere	362	100.0	306	84.5	36	10.0	13	3.6	7	1.9
7-00 Production supervisors and general foremen	330	100.0	275	83.4	35	10.6	11	3.3	9	2.7
8-43 Motor-Vehicle Mechanics	260	100.0	221	85.0	29	11.1	7	2.7	3	1.2
8-71 Plumbers and Pipe Fitters	244	100.0	190	77.9	39	16.0	10	4.1	5	2.0
8-73 Sheet-Metal Workers	241	100.0	205	85.1	25	10.4	8	3.3	3	1.2
9-99 Labourers not elsewhere classified	232	100.0	190	81.9	26	11.2	10	4.3	6	2.6
8-55 Electrical Wiremen	179	100.0	151	84.4	17	9.5	7	3.9	4	2.2
8-34 Machine-Tool Operators	166	100.0	135	81.3	20	12.1	6	3.6	5	3.0
8-72 Welders and flame-Cutters	164	100.0	134	81.7	17	10.4	7	4.2	6	3.7
8-41 Machinery Fitters and Machine Assemblers	163	100.0	142	87.1	14	8.6	5	3.1	2	1.2

Table S4. Most represented industries (ISIC Rev 2) in all subjects and according interstitial abnormalities detected at CT.

	All Subjects (*n*=2,157)		Absent or Gravity-Dependant Opacities (*n*=1,794)		Minor Interstitial Abnormalities (*n*=226)		Interstitial Abnormalities Inconsistent with UIP (*n*=82)		UIP Pattern or Possible UIP Pattern (*n*=55)	
	n	%	*n*	%	*n*	%	*n*	%	*n*	%
5000 Construction	893	100.0	731	81.9	93	10.4	45	5.0	24	2.7
3710 Iron and steel basic industries	403	100.0	328	81.4	42	10.4	25	6.2	8	2.0
3813 Manufacture of structural metal products	324	100.0	265	81.8	39	12.0	12	3.7	8	2.5
9513 Repair of motor vehicles and motorcycles	274	100.0	231	84.3	31	11.3	8	2.9	4	1.5
3843 Manufacture of motor vehicles	211	100.0	180	85.3	17	8.1	4	1.9	10	4.7
3824 Manufacture of special industrial machinery and equipment [except metal and wood working machinery	190	100.0	159	83.7	22	11.6	5	2.6	4	2.1
3841 Ship building and repairing	187	100.0	155	82.9	24	12.8	5	2.7	3	1.6
1110 Agriculture and livestock production	130	100.0	102	78.5	15	11.5	6	4.6	7	5.4
6200 Retail Trade	127	100.0	106	83.5	12	9.5	4	3.1	5	3.9
3699 Manufacture of non-metallic mineral products not elsewhere classifies	109	100.0	90	82.6	11	10.0	4	3.7	4	3.7
